# Medical devices preparation model development for quality services in Srinagarind Hospital Faculty of Medicine, Khon Kaen University, Thailand

**DOI:** 10.1017/ash.2025.137

**Published:** 2025-09-03

**Authors:** Sasithorn Ruangprasertkul, Ponsawan Quobuwan, Gesorn Sittisart, Amara Sirithongsuk, Nisachon Rukkwoamsue, Waraporn Wiangwiset

**Affiliations:** 1 Regional Nurse: RN; 2 Deputy Technicians, Srinagarind Hospital, Faculty of MedicineKhon Kaen University, Thailand

## Abstract

**Background and objective:** Preparation of medical devices is a critical component of healthcare services. Used reusable items require reprocessing and sterilization, which eliminate contaminant microorganisms, to ensure safety and availability prior to subsequent applications. The objective of this research is to develop medical device preparation guidelines which address existing quality issues for quality services. **Methods:** This action research was conducted in three phases. Phase one, research planning, literature review of APSICS guideline and work system development techniques 5M model. Phase Two included guideline implementation, observation and analysis. In the 1^st^ year, staff members were trained to perform duties according to the new guidelines, new inspection categories were added, and cleaning procedures were improved. In the 2^nd^ year, new policies for collection of used items were implemented, ATP test was introduced to improve microorganism detection. The team oversaw more staff training and quality control of RO water used in cleaning medical devices in the 3^rd^ year. In the 4^th^ year, more staff members were trained and water supply pipes were replaced to accommodate increasing RO water demands. Trained staff members received follow- ups and percentages of reprocessed medical items that passed quality criteria were monitored. Phase three was analysis and evaluation, using the number of staff members who received training, percentages of reprocessed medical items that passed quality criteria. **Results:** The number of staff members who performed tasks correctly increased every year. The percentage of reprocessed reusable medical items which met quality requirements from 2018 - 2022 showed a steady increase (R^2^ = 0.860) (91.82, 95.33, 98.95, 98.68 and 99.42, respectively).The score for compliance to APSICS guideline for cleaning & decontamination processes from 2020 - 2022 increased continuously (78.57,96.43, and 100.00). **Conclusion:** The approaches to medical device preparation align with 5M model requiring proper management for maximum efficiency and cost effectiveness.

